# To Subscribers

**Published:** 1860

**Authors:** 


**To Subscribers.**

We shall shortly send out the bills of those indebted for the Journal, hoping to receive a prompt response. Should any mistakes occur, we hope to be excused, and will promptly rectify, upon being informed of the fact. The books have recently changed hands, and as the result, some confusion and error may be readily anticipated, but in any such case the statement of the individual, to whom an account is sent, will be sufficient to insure a correction. Should any of our friends be overlooked, we hope they will not wait to be reminded of their delinquency.

